# *KRAS*, *NRAS*, and *BRAF* mutations are highly enriched in trisomy 12 chronic lymphocytic leukemia and are associated with shorter treatment-free survival

**DOI:** 10.1038/s41375-019-0444-6

**Published:** 2019-03-14

**Authors:** Elena Vendramini, Riccardo Bomben, Federico Pozzo, Dania Benedetti, Tamara Bittolo, Francesca Maria Rossi, Michele Dal Bo, Kari G. Rabe, Gabriele Pozzato, Francesco Zaja, Annalisa Chiarenza, Francesco Di Raimondo, Esteban Braggio, Sameer A. Parikh, Neil E. Kay, Tait D. Shanafelt, Giovanni Del Poeta, Valter Gattei, Antonella Zucchetto

**Affiliations:** 1 Clinical and Experimental Onco-Hematology Unit, Centro di Riferimento Oncologico di Aviano (CRO) IRCCS, Aviano (PN), Italy; 2 Division of Biomedical Statistics and Informatics, Department of Health Sciences Research, Mayo Clinic, Rochester, MN USA; 3 Department of Internal Medicine and Haematology, Maggiore General Hospital, University of Trieste, Trieste, Italy; 4 Division of Hematology, Ferrarotto Hospital, Catania, Italy; 5 Division of Hematology, Department of Medicine, Mayo Clinic, Rochester, MN USA; 6 Department of Hematology/Oncology, Stanford University, Stanford, CA USA; 7 Division of Hematology, S. Eugenio Hospital and University of Tor Vergata, Rome, Italy

**Keywords:** Chronic lymphocytic leukaemia, Cancer genetics

## To the Editor

*KRAS* mutations are among the most common oncogenic events in human carcinomas of endodermal origin, whose presence predicts for resistance to several target therapies [1]. Conversely, little is known regarding the role and/or clinical impact of *KRAS* mutations in the setting of the hematological malignancies, including chronic lymphocytic leukemia (CLL), and only in recent years extensive sequencing data have highlighted the recurrent mutations of genes affecting the Ras–MAPK pathway in CLL [2, 3]. These mutations, by leading to a constitutive activation of MAPK signaling pathway, have emerged as relevant in driving impaired clinical responses to lenalidomide and chlorambucil, and acquired resistance to fludarabine as well as to PI3K and BCL2 inhibitors [4–6]. In this context, some studies pinpointed a higher frequency of mutations in members of the Ras–MAPK pathway in CLL cases with specific clinico-biological features [6, 7], including the presence of trisomy 12, a cytogenetic aberration associated with a unique pathophysiology among CLL [8, 9], and/or an unmutated (UM) configuration of *IGHV* genes, although a dedicated and comprehensive analysis of these aspects is still missing.

This study, approved by the IRB of the Aviano Centro di Riferimento Oncologico (Approvals n. IRB-05-2010 and n. IRB-05-2015), included 534 primary CLL from treatment-naive patients. The cohort was purposely enriched in trisomy 12 CLL by including 110 cases from the Mayo Clinic, Rochester, MN [8] to better evaluate the incidence of mutations of the Ras–MAPK pathway in these subsets. Overall, out of 534 cases, trisomy 12 CLL accounted for 300 cases (190 with trisomy 12 as the sole abnormality [trisomy 12-only], and 110 with trisomy 12 plus another abnormality on FISH [trisomy 12-plus]), 332 cases had UM *IGHV* genes, and 214 cases had *NOTCH1* aberrations (details in Table S1). CLL patients were diagnosed and treated according to the current iwCLL 2018 guidelines [10], and all samples were collected at diagnosis from treatment-naive patients. In 442/534 cases (clinical cohort), treatment-free survival (TFS) data were available along with a comprehensive clinical and biological characterization (Table S1 and Supplemental Methods). This cohort showed the expected clinical behavior according to both the stratification of the established cytogenetic categories and to the canonical prognosticators by univariable and multivariable analyses (Supplemental Figure S1 and Table S2). Mutation testing for *KRAS*, *NRAS, BRAF, TP53, NOTCH1*, *BIRC3*, and *SF3B1* was performed on DNA from CD19^+^ enriched CLL samples by Next Generation Sequencing (NGS) assays with at least 1000 × coverage and 1% sensitivity (details in Supplemental Methods). Groups were compared by chi-square test; TFS was computed from diagnosis to treatment and analyzed by log-rank test and Cox regression analysis with a stepwise procedure using MedCalc Statistical Software version 16.8.4 (MedCalc Software bvba, Ostend, Belgium; https://www.medcalc.org; 2016).

**Fig. 1:**
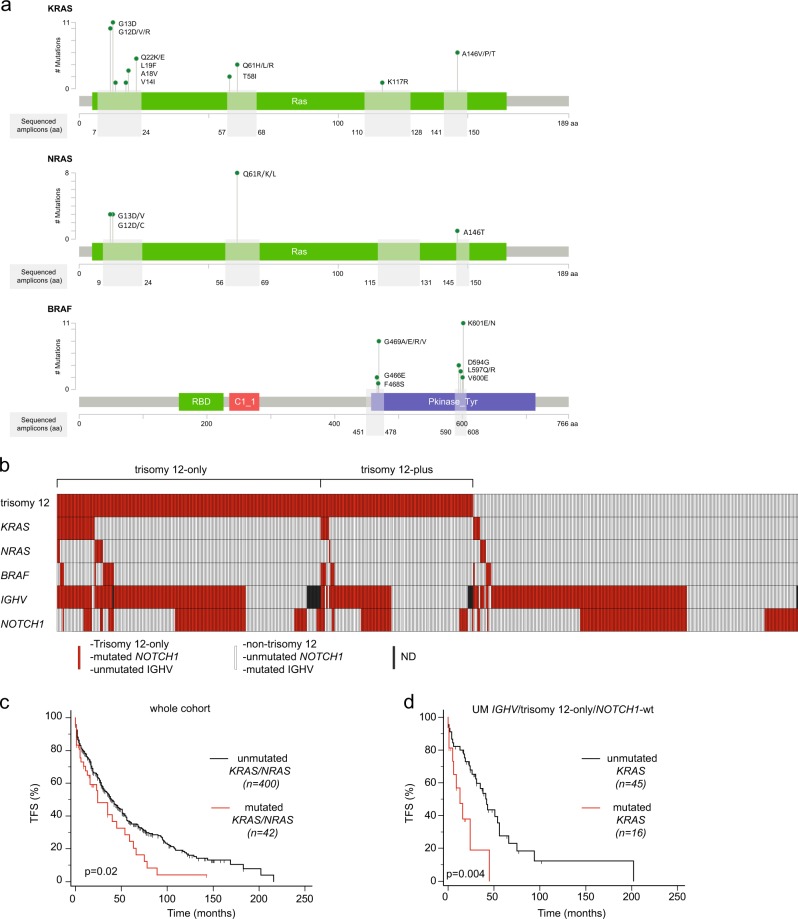
Type, incidence and prognostic impact of *KRAS*, *NRAS* and *BRAF* mutations. **a** Lollipop plots of mutations found in *KRAS*, *NRAS,* and *BRAF* genes. Sites and frequency of missense point mutations, and schematic presentation of the protein structure and functional domains are shown (MutationMapper, cBioPortal Version 1.14.0, Gao et al. Sci. Signal. 2013 and Cerami et al. Cancer Discov. 2012). Gray boxes indicate amino acids (aa) regions corresponding to the sequenced amplicons. *RBD*, Ras-binding domain; *C1_1*, phorbol esters/diacylglycerol binding domain (C1 domain); *Pkinase_Tyr*, protein tyrosine kinase domain. **b** Co-mutation plot of 534 CLL analyzed for *KRAS*, *NRAS*, *BRAF* mutations. Incidence of trisomy 12, *KRAS*, *NRAS,* and *BRAF* missense mutations, *NOTCH1* aberrations and *IGHV* status are shown. **c** Kaplan–Meier curves of treatment-free survival (TFS) of 442 CLL patients stratified by the presence of *KRAS* and/or *NRAS* mutations. **d** Kaplan–Meier curves of TFS of 61 CLL patients in the *IGHV* unmutated/trisomy 12-only/*NOTCH1*-wt group stratified by the presence of *KRAS* mutations

The mutation analysis of the Ras–MAPK pathway was focused on the *KRAS*, *NRAS*, and *BRAF* genes, previously reported as the most frequently mutated genes among the members of the pathway [2]. We found 91 missense point mutations in 64 CLL cases, with a prevalence of *KRAS* (44 mutations in 38 [7.1%] patients), followed by *BRAF* (32 mutations in 24 [4.5%] patients) and *NRAS* (15 mutations in 13 [2.4%] patients). Nearly all mutations were previously associated with the gain-of-function phenotype and increased RAS/ERK downstream signaling (Fig. 1a and Table S3) [1]. In particular, among the most frequent *KRAS*/*NRAS* mutations, almost half of the mutations (27/59, 45%), overall affecting 23/49 (47%) patients, involved the G12/G13 codons, in keeping with what was observed in colon and lung cancers (Table S3) [1]. The co-occurrence of 2 mutated genes was observed in 11 cases (*KRAS* and *BRAF* in 8/11 cases, *KRAS* and *NRAS* in 2/11 cases, *NRAS* and *BRAF* in 1/11 cases), whereas mutations affecting all three genes were not found in our cohort. The mutations were mainly subclonal (mean Variant Allele Fraction, VAF, 12.3%, range 1.3–61.6%) with one-third of mutations (33/91) above 10% VAF. The presence of multiple mutations affecting the same gene occurred in 14 cases, including 5 cases that presented mutations in the same or adjacent codons (i.e., one case with both K601N and K601E *BRAF* mutations, one case with V600E and K601E *BRAF* mutations, and three cases with two simultaneous *KRAS* mutations at the G12 and G13 codons) suggesting that multiple genetic hits are positively selected in different subclones within the same leukemia specimen.

A strong association between *KRAS*/*NRAS*/*BRAF* mutations and the presence of an UM *IGHV* gene status and trisomy 12 was observed (Fig. 1b and Table S4). Overall, 87.3% of *KRAS*/*NRAS*/*BRAF* mutated cases had UM *IGHV* (*p* < 0.0001) and 79.7% were trisomy 12 CLL (*p* < 0.0001). Concordantly, the highest *KRAS*/*NRAS*/*BRAF* mutation frequency was found in CLL patients with concomitant UM *IGHV* and trisomy 12-only (38/133, 28.6%). This group was characterized by 25 *KRAS* (18.8%), 8 *NRAS* (6%) and 11 *BRAF* (8.3%) mutated cases. Of note, the UM *IGHV*/trisomy 12-only group was characterized by a higher frequency of *KRAS*/*NRAS*/*BRAF* mutations also when compared to the UM *IGHV*/trisomy 12-plus group (8/49, 16.3%). Finally, the lowest frequency of *KRAS/NRAS/BRAF* mutations was observed in the context of CLL patients with M *IGHV* (8/186, 4.3%) and del13q as the sole chromosomal aberration (2/94, 2.1% in the whole cohort, and 2/53, 3.8% in the context of UM *IGHV* cases).

We then correlated the presence of *KRAS*/*NRAS*/*BRAF* mutations to other biological features (Table S4). When considering the whole CLL cohort, the only variables associated with a higher frequency of *KRAS*/*NRAS*/*BRAF* mutations were the absence of *BIRC3* mutations (*p* = 0.02) and the positive expression (≥30%) of CD49d (*p* = 0.04). On the other hand, if circumscribing the analysis to UM *IGHV*/trisomy 12 CLL, CD49d positive expression lost its association with *KRAS*/*NRAS*/*BRAF* mutations, as expected due to the almost universal CD49d expression in trisomy 12 CLL patients [9]. Conversely, we observed a higher frequency of *KRAS*/*NRAS*/*BRAF* mutations in *NOTCH1* wild type cases (29/92, 31.5%) and *BIRC3* wild type cases (41/132, 31.1%) compared to their mutated counterparts (*NOTCH1* mutated: 17/90, 18.9%; *BIRC3* mutated: 4/30, 13.3%; *p* = 0.05 in both cases), pointing to a mutual exclusivity of these mutations in the pathogenesis of the disease. No other significant associations with other known prognostic variables such as presence of *TP53* mutations/disruption, *SF3B1* mutations, ZAP-70 and CD38 expression, Rai staging, age at diagnosis, and gender were observed either in the whole cohort or in the UM *IGHV*/trisomy 12 cohort (Table S4).

We finally evaluated the prognostic relevance of *KRAS*, *NRAS*, and *BRAF* mutations as predictors of TFS. In the context of the clinical cohort, the presence of either *KRAS* or *NRAS* mutations or the concomitant presence of *KRAS/NRAS* mutations were associated with shorter TFS (*p* = 0.07, *p* = 0.05, and *p* = 0.02, respectively) (Table 1, Fig. 1c). Conversely, *BRAF* mutations were not associated with TFS, pointing to a secondary role of BRAF in the Ras–MAPK pathway in CLL, in line with studies indicating the lack of therapeutic effects of BRAF inhibition in CLL [11]. In a multivariable model that included the main known CLL prognosticators, the presence of *KRAS*/*NRAS* mutations retained its independent prognostic power as predictor for shorter TFS (*p* = 0.03, Table 1). Moreover, circumscribing the analysis to the CLL subgroup with the highest incidence of these mutations (i.e., UM *IGHV*/trisomy 12-only/*NOTCH1-*wt), both *KRAS* mutations alone (*p* = 0.005) and *KRAS*/*NRAS* mutations (*p* = 0.05) were associated with shorter TFS (Fig. 1d and Table S5), and the presence of *KRAS* mutations retained its prognostic value in a multivariable analysis that included all the variables with an impact in univariable analysis (*p* = 0.01, Table S5). The subclonal or clonal pattern of *KRAS/NRAS* mutations had similar negative impact in our series (not shown), as previously observed for other gene mutations in CLL [12], and in keeping with the known capability of *KRAS* mutated tumor cells to enhance the overall tumor cell fitness by influencing the non-mutated neoplastic component [13].

**Table 1:** Cox regression analysis of treatment-free survival in the whole cohort

		Univariable		Multivariable (*n* = 365)	
	N pts analyzed	HR (95% CI)	*p*-value	HR (95% CI)	*p*-value
male gender	442	0.97 (0.76–1.22)	0.8	−	−
age ≥ 65	441	0.97 (0.77–1.23)	0.8	−	−
Rai stage II–III–IV	434	2.60 (2.03–3.32)	<0.0001	2.59 (1.98–3.41)	<0.0001
CD49d positive (≥30%)	440	1.48 (1.13–1.92)	0.004	1.65 (1.22–2.23)	0.001
CD38 positive (≥30%)	439	1.13 (0.89–1.43)	0.3	−	−
ZAP-70 positive (≥20%)	389	1.44 (1.11–1.87)	0.005	n.i.	n.i.
I*GHV* unmutated	428	2.16 (1.66–2.8)	<0.0001	1.83 (1.37–2.45)	<0.0001
*TP53* disrupted (del17p and/or TP53 mutated)	442	1.70 (1.24–2.32)	0.0008	1.46 (1.05–2.03)	0.024
*NOTCH1* mutated	442	1.37 (1.08–1.73)	0.009	n.i.	n.i.
*SF3B1* mutated	327	1.46 (0.90–2.38)	0.1	−	−
*BIRC3* mutated	343	0.93 (0.65–1.34)	0.7	−	−
*KRAS* mutated	442	1.49 (0.96–2.30)	0.073	−	−
*NRAS* mutated	442	1.86 (0.99–3.50)	0.055	−	−
*BRAF* mutated	442	1.34 (0.75–2.40)	0.3	−	−
*KRAS/NRAS* mutated	442	1.54 (1.05–2.25)	0.025	1.56 (1.04–2.36)	0.033

Factors with *p*-value <0.05 in univariable analysis were entered in the multivariable analysis

*HR* hazard ratio, *CI* confidence interval, *n.i.* variables not included in the model after stepwise selection

In the present study, we demonstrated that *KRAS*, *NRAS*, and *BRAF* mutations were almost exclusively found in UM *IGHV*/trisomy 12 CLL and were almost mutually exclusive with *NOTCH1* and *BIRC3* mutations. The type of genomic structural variants, especially trisomy 12 and del13q, strongly influenced *KRAS*/*NRAS*/*BRAF* mutation incidence, that turned out to be at the highest level in cases bearing trisomy 12 as the sole genomic aberration, intermediate in cases in which trisomy 12 was associated with other genetic aberrations, mainly del13q, and at the lowest level in cases bearing del13q as the sole FISH detectable genetic aberration. This peculiar distribution of *KRAS*/*NRAS*/*BRAF* mutation incidence is in keeping with a CLL pathogenetic model in which the two main founder genetic lesions (i.e., trisomy 12 and del13q) identify CLL subgroups following different patho-biological pathways. In particular, the presence of del13q, given its link to the miR15/miR16-BCL2 axis, characterizes a CLL subset especially oriented toward the amplification of anti-apoptotic signals [14]. On the other hand, in trisomy 12 CLL, the co-presence of *KRAS*/*NRAS*/*BRAF* mutations and/or *NOTCH1* mutations and/or *BIRC3* mutations along with a UM *IGHV* gene status and over-expression of surface receptors mediating microenvironment interactions (e.g., CD49d) more likely characterizes CLL with amplified pro-survival and proliferative signals [8, 9, 15]. This may explain the clinical association between *KRAS*/*NRAS* mutations and shorter TFS, as shown in the present analysis.

Given the reported high risk of poor response and development of chemo-resistance characterizing CLL cases with *KRAS*/*NRAS* mutations [4–6], additional therapeutic strategies should be considered for the treatment of these cases, including MEK/ERK inhibitors, employed alone or in combination with conventional therapies.

## Supplementary information

Supplemental Material
