# Supplementary material for: KRAS, NRAS, and BRAF mutations are highly enriched in trisomy 12 chronic lymphocytic leukemia and are associated with shorter treatment-free survival
Source: Leukemia. 2019 Mar 14;33(8):2111–5. doi: 10.1038/s41375-019-0444-6 (PMC6756038; doi:10.1038/s41375-019-0444-6)

Supplemental Materials for

KRAS, NRAS, and *BRAF* Mutations Are Highly Enriched in Trisomy 12 Chronic Lymphocytic Leukemia and Are Associated With Shorter Treatment-Free Survival

Supplemental information:

•**Supplemental Material and Methods**

• **Supplemental Tables:**

o Table S1. Clinical and laboratory features of CLL patients.

o Table S2. Cox regression analysis of treatment-free survival of the clinical cohort.

o Table S3. List of *KRAS, NRAS,* and *BRAF* missense point mutations identified in the study cohort.

o Table S4. Association of *KRAS, NRAS,* and *BRAF* mutations with CLL clinico-biological features.

o Table S5. Cox regression analysis of treatment-free survival in the *IGHV* unmutated/trisomy 12-only/*NOTCH1*-wt subgroup.

• **Supplemental Figure Legend**

• **Supplemental Figure:**

o Figure S1. Kaplan-Meier curves of treatment-free survival of CLL patients in the clinical cohort.

Supplemental Materials and Methods

Patient characterization

Patient characterization included Rai staging, expression of CD49d (CD49d positive: ≥30%), CD38 (CD38 positive: ≥30%) and ZAP-70 (ZAP-70 positive: ≥20%) and *IGHV* mutational status (UM if < or equal to 2% mutations from germline sequence). Interphase fluorescent *in situ* hybridization (FISH) on peripheral blood investigated trisomy12, del13q, del11q, del17p; cases bearing each aberration in ≥ 10% of nuclei were considered abnormal.

Mutational analysis by targeted next-generation sequencing

KRAS, *NRAS*, *BRAF*, *TP53* and *NOTCH1* mutational status was investigated by next generation sequencing (NGS) with an amplicon based strategy. Specific primers for *KRAS*, *NRAS* (hotspot regions in exons 2, 3 and 4), *BRAF* (hotspot regions in exons 11 and 15), *TP53* (exons 2-11) and *NOTCH1* (exon 34 including 3'UTR), were designed with the ION Ampliseq designer (Thermo Scientific, Milan, Italy, https://www.ampliseq.com/), checked using the Primer3 program (1;2), and modified according to Illumina (San Diego, CA) protocol adding specific adapter sequences (see (3)). Amplicon libraries were generated using a modified Illumina protocol. Briefly, multiplex PCR products were generated using Phusion High-Fidelity DNA Polymerase (Thermo Scientific) and subsequently tagged with specific index according to modified procedures for NexteraXT (DNALibrary Preparation kit (Illumina). *BIRC3* and *SF3B1* mutational status was investigated by NGS using an amplicon based strategy coupled with NexteraXT (Illumina). Specific primers for *BIRC3* (exons 6-9) and *SF3B1* (exons 14-18), were designed with the Primer3 program (1;2). Briefly, multiplex PCR products were generated using Phusion High-Fidelity DNA Polymerase (Thermo Scientific) and subsequently processed and indexed with NexteraXT kit according to manufacter's instructions. Purified libraries were pooled, and paired-end sequenced in a MiSeq instrument (Illumina). The sequencing coverage was at least 1000X.

Bioinformatic workflow

Sequencing reads were mapped to the human reference genome (GRCh37/hg19) using the Burrows-Wheeler Aligner–MEM algorithm (version 0.7.10) (4). Coverage along the targeted regions was analyzed using SAMtools (version 1.1) (4). Variant calling was performed using the entire pipeline established on the MiSeq Reporter software (MSR; version 2.4.60). Data were analyzed with MiSeq reporter (Illumina) and IGV software (5;6) against human genome assembly GRCh37/hg19. Results

were expressed as percentage of mutated DNA. Cases with >1% mutated DNA were considered mutated. Synonymous variants and polymorphisms described in the Single Nucleotide Polymorphism Database (dbSNP138) removed. All other variants were considered as somatic mutations when they were truncating, affected splicing sites, or were identified as somatic mutations in COSMIC (http://cancer.sanger.ac.uk/cosmic), the InternationalAgency for Research on Cancer TP53 database (http://p53.iarc.fr/), or The Clinical Knowledgebase (CKB) database (https://ckb.jax.org/).

References to supplemental Materials and Methods

1. Koressaar T, Remm M. Enhancements and modifications of primer design program Primer3. Bioinformatics. 2007;23:1289-91.

2. Untergasser A, Cutcutache I, Koressaar T, Ye J, Faircloth BC, Remm M, et al. Primer3--new capabilities and interfaces. Nucleic Acids Res. 2012;40:e115.

3. D'Agaro T, Bittolo T, Bravin V, Dal BM, Pozzo F, Bulian P, et al. NOTCH1 mutational status in chronic lymphocytic leukaemia: clinical relevance of subclonal mutations and mutation types. Br J Haematol. 2018;182:597-602.

4. Li H, Durbin R. Fast and accurate short read alignment with Burrows-Wheeler transform. Bioinformatics. 2009;25:1754-60.

5. Robinson JT, Thorvaldsdottir H, Winckler W, Guttman M, Lander ES, Getz G, et al. Integrative genomics viewer. Nat Biotechnol. 2011;29:24-6.

6. Thorvaldsdottir H, Robinson JT, Mesirov JP. Integrative Genomics Viewer (IGV): high-performance genomics data visualization and exploration. Brief Bioinform. 2013;14:178-92.

Table S1. Clinical and laboratory features of CLL patients.

Parameter	n/total cases	%
Gender, male	317/534	59.4
Age median, years [range]	64 [31-89]	
≥65	210/447	47.0
IGHV unmutated	332/518	64.1
Chromosomal aberrations		
trisomy 12	300/534	56.2
trisomy 12-only	190/534	35.6
trisomy 12-plus	110/534	20.6
del13q	71/534	13.3
del11q	17/534	3.2
del17p	14/534	2.6
del13q and del11q	3/534	0.6
del13q and del17p	5/534	0.9
Non-trisomy 12	234/534	43.8
del13q	94/534	17.6
normal	82/534	15.4
del11q	16/534	3.0
del17p	13/534	2.4
other	29/534	5.4
NOTCH1 mutated	214/534	40.1
SF3B1 mutated	30/379	7.9
BIRC3 mutated	69/393	17.6
TP53 mutated	59/534	11.0
CD49d positive (≥30%)	373/531	70.2
CD38 positive (≥30%)	285/530	53.8
ZAP-70 positive (≥20%)	278/465	59.8
Rai stage		
0-I	315/439	71.8
II-III-IV	124/439	28.2
Treated	290/442	65.6

Trisomy 12-only: cases harboring trisomy 12 as the sole chromosomal aberration; trisomy 12-plus: cases harboring trisomy 12 plus other chromosomal aberrations (listed); other: non-trisomy 12 cases harboring multiple chromosomal aberrations among those tested (i.e. del13q and/or plus del11q and/or plus del17p).

Table S2. Cox regression analysis of treatment-free survival of the clinical cohort.

	Multivariable (n=365)	
	HR (95% CI)	p-value
I*GHV* unmutated	1.89 (1.41-2.52)	<0.0001
TP53 disrupted (del17p and/or *TP53* mutated)	1.41 (1.01-1.95)	0.04
CD49d positive (≥30%)	1.66 (1.23-2.24)	0.001
Rai stage II-III-IV	2.54 (1.94-3.33)	<0.0001

Multivariable Cox regression analysis was performed by including factors with p-value <0.05 in univariable analysis: *IGHV*, *NOTCH1*, CD49d, *TP53* disrupted, del11q (Döhner classification), ZAP-70 and Rai stage; HR: hazard ratio; CI: confidence interval. Results are based on the final model after stepwise selection of covariates.

Table S3. List of *KRAS*, *NRAS*, and *BRAF* missense point mutations identified in the study cohort.

Case #	*KRAS*		*NRAS*		*BRAF*	
	sequence variation	VAF (%)	sequence variation	VAF (%)	sequence variation	VAF (%)
1	c.436G>A p.A146T	23.3			c.1790T>A p.L597Q	9.0
2	c.53C>T p.A18V	18.8				
3	c.436G>C p.A146P	3.0				
4	c.436G>C p.A146P	31.2				
5	c.437C>T p.A146V	5.7				
6	c.437C>T p.A146V	6.3			c.1801A>G p.K601E	9.0
7	c.437C>T p.A146V	8.6				
8	c.35G>A p.G12D; c.38G>A p.G13D; c.173C>T p.T58I	1.8; 2.8; 2.8	c.182A>G p.Q61R	1.6		
9	c.35G>A p.G12D; c.38G>A p.G13D	2.0; 3.4	c.38G>A p.G13D	1.8		
10	c.35G>A p.G12D	2.8				
11	c.35G>A p.G12D	26.2				
12	c.35G>A p.G12D	32.3				
13	c.34G>C p.G12R	15.1				
14	c.35G>T p.G12V	12.7				
15	c.35G>T p.G12V	5.3				
16	c.35G>T p.G12V; c.38G>A p.G13D	7.9;3.5				
17	c.38G>A p.G13D	4.6			c.1803A>T p.K601N; c.1790T>G p.L597R	5.3; 4.0
18	c.38G>A p.G13D	1.3				
19	c.38G>A p.G13D	2.2			c.1801A>G p.K601E	24.0
20	c.38G>A p.G13D; c.182A>T p.Q61L	23.9; 3.3				
21	c.38G>A p.G13D	28.4				
22	c.38G>A p.G13D	33.6				
23	c.38G>A p.G13D	5.7				
24	c.38G>A p.G13D	6.6				

25	c.350A>G p.K117R					
26	c.57G>T p.L19F	3.3			c.1803A>T p.K601N; c.1801A>G p.K601E ; c.1790T>A p.L597Q	8.6; 3.3; 13.5
27	c.57G>T p.L19F	39.9				
28	c.57G>T p.L19F	56.8				
29	c.64C>G p.Q22E[a]	2.2			c.1781A>G p.D594G	4.4
30	c.64C>A p.Q22K	12.4				
31	c.64C>A p.Q22K	14.8			c.1406G>C p.G469A	5.9
32	c.64C>A p.Q22K	20.4				
33	c.64C>A p.Q22K	8.1				
34	c.183A>C p.Q61H	31.2				
35	c.183A>T p.Q61H	6.3				
36	c.182A>G p.Q61R; c.35G>A p.G12D	2.0; 1.6				
37	c.173C>T p.T58I	20.7				
38	c.40G>A p.V14I	8.8			c.1803A>T p.K601N	16.8
39			c.436G>A p.A146T	2.1		
40			c.34G>T p.G12C	4.1		
41			c.35G>A p.G12D; c.181C>A p.Q61K	3.1; 23.3		
42			c.35G>A p.G12D	43.2		
43			c.38G>T p.G13V	22.7		
44			c.181C>A p.Q61K	1.5		
45			c.181C>A p.Q61K; c.38G>A p.G13D	5.9; 27.3		
46			c.182A>T p.Q61L	1.5		
47			c.182A>G p.Q61R	1.8	c.1406G>T p.G469V; c.1397G>A p.G466E	10.6; 5.0
48			c.182A>G p.Q61R	28.1		
49			c.182A>G p.Q61R	8.3		
50					c.1781A>G p.D594G	2.6
51					c.1781A>G p.D594G; c.1406G>C p.G469A	3.7; 3.7

52					c.1781A>G p.D594G	6.1
53					c.1403T>C p.F468S	3.6
54					c.1397G>A p.G466E	25.3
55					c.1406G>C p.G469A	31.9
56					c.1406G>C p.G469A	61.6
57					c.1406G>A p.G469E	1.6
58					c.1405G>C p.G469R	34.1
59					c.1801A>G p.K601E	19
60					c.1801A>G p.K601E; c.1799T>A p.V600E	3.2; 29.1
61					c.1801A>G p.K601E; c.1406G>C p.G469A	8.0; 4.6
62					c.1803A>T p.K601N	1.6
63					c.1803A>T p.K601N	7.7
64					c.1799T>A p.V600E; c.1406G>C p.G469A	2.6; 17.6

Variations in the coding (c) and protein (p) sequence are annotated. VAF: Variant Allele Fraction. [a]Not in COSMIC database (https://cancer.sanger.ac.uk/cosmic) but reported in The Clinical Knowledgebase (CKB) database (https://ckb.jax.org/).

Table S4. Association of *KRAS*, *NRAS*, and *BRAF* mutations with CLL clinico-biological features.

		All cohort				UM *IGHV*/trisomy 12 cohort			
		Total	mutated *KRAS-NRAS-BRAF*	unmutated *KRAS-NRAS-BRAF*	p-value	Total	mutated *KRAS-NRAS-BRAF*	unmutated *KRAS-NRAS-BRAF*	p-value
IGHV	unmutated	332	55	277	<0.0001	-	-	-	-
	mutated	186	8	178		-	-	-	
STRUCTURAL VARIANT	trisomy 12	300	51	249	<0.0001	-	-	-	-
	non-trisomy 12	234	13	221		-	-	-	
STRUCTURAL VARIANT	trisomy 12-only	190	41	149	0.005	133	38	95	0.1
	trisomy 12-plus	110	10	100		49	8	41	
NOTCH1	unmutated	320	43	277	0.2	92	29	63	0.05
	mutated	214	21	193		90	17	73	
SF3B1	unmutated	349	50	299	0.4	145	41	104	1
	mutated	30	6	24		14	4	10	
BIRC3	unmutated	324	55	269	0.02	132	41	91	0.05
	mutated	69	4	65		30	4	26	
TP53	normal	460	55	405	0.9	162	42	120	0.6
	Disrupted (del17p and/or *TP53* mutated)	74	9	65		20	4	16	
CD49d	negative	158	12	146	0.04	23	6	17	0.9
	positive (≥30%)	373	52	321		158	40	118	
CD38	negative	245	27	218	0.2	70	17	53	0.8
	positive (≥30%)	285	37	248		111	29	82	
ZAP-70	negative	187	18	169	0.2	34	7	27	0.5
	positive (≥20%)	278	39	239		131	34	97	

Rai stage	0-I	315	42	273	0.5	114	32	82	0.4
	II-III-IV	124	14	110		38	8	30	
age	<65	237	24	213	0.07	73	17	56	0.4
	≥65	210	33	177		84	24	60	
gender	male	317	37	280	0.8	115	26	89	0.3
	female	217	27	190		67	20	47	

The association between recurrent mutations and the other clinical and biological prognosticators was calculated using the chi-square test. Trisomy 12-only: cases harboring trisomy 12 as the sole chromosomal aberration; trisomy 12-plus: cases harboring trisomy 12 plus other chromosomal aberrations (del13q = 71 cases; del11q = 17 cases; del17p = 14 cases; del13q and del17p = 5 cases; del13q and del11q = 3 cases); non-trisomy 12: cases harboring other chromosomal aberrations than trisomy 12 or none.

Table S5. Cox regression analysis of treatment-free survival in the *IGHV* unmutated/trisomy 12-only/*NOTCH1*-wt subgroup.

		Univariable		Multivariable (n=57)	
	N pts analyzed	HR (95% CI)	p-value	HR (95% CI)	p-value
KRAS mutated	61	2.99 (1.36-6.57)	0.006	2.88 (1.28-6.48)	0.01
NRAS mutated	61	1.31 (0.51-3.44)	0.6	−	−
BRAF mutated	61	0.92 (0.28-3.00)	0.9	−	−
KRAS/NRAS mutated	61	1.97 (1-3.89)	0.05	−	−
SF3B1 mutated	60	2.92 (1.19-7.17)	0.02	n.i.	n.i.
BIRC3 mutated	60	0.69 (0.27-1.76)	0.4	−	−
TP53 disrupted (del17p and/or *TP53* mutated)	61	1.47 (0.20-10.91)	0.7	−	−
CD49d positive(≥30%)	61	1.30 (0.46-3.66)	0.6	−	−
CD38 positive (≥30%)	61	0.59 (0.31-1.11)	0.1	−	−
ZAP-70 positive (≥20%)	58	0.99 (0.48-2.01)	1.0	−	−
Rai stage II-III-IV	58	2.77 (1.28-5.98)	0.01	2.70 (1.24-5.88)	0.012
age ≥65	61	1.52 (0.78-2.96)	0.2	−	−

Factors with p-value <0.05 in univariable analysis were entered in the multivariable analysis; HR: hazard ratio; CI: confidence interval; n.i.: variables not included in the model after stepwise selection.

Supplemental Figure Legend

Figure S1. Kaplan-Meier curves of treatment-free survival of CLL patients in the clinical cohort. Kaplan-Meier curves of treatment-free survival of CLL patients stratified by the presence of main CLL prognosticators: Döhner hierarchical cytogenetic classification (442 cases); *TP53* disruption (del17p and/or *TP53* mutated, 442 cases); *IGHV* mutational stauts (428 cases); CD49d expression (positive ≥30%, 442 cases); Rai stage (434 cases); *NOTCH1* mutational status (442 cases); ZAP-70 expression (positive ≥20%, 389 cases).

Supplemental Figure 1

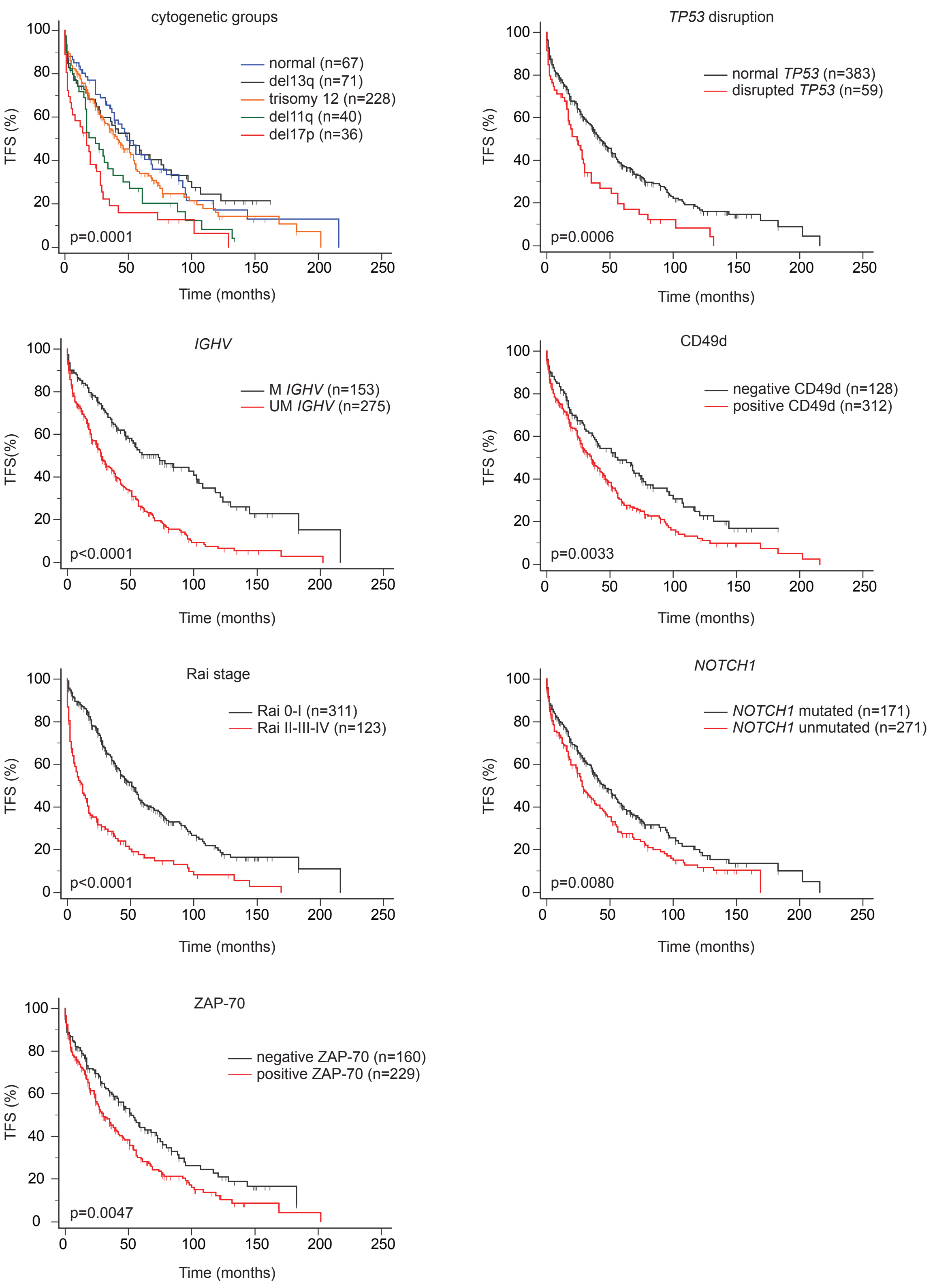

Supplement: Supplementary file 1 — Supplemental Material [file 41375_2019_444_MOESM1_ESM.pdf]
